# Fatty-Acid Uptake in Prostate Cancer Cells Using Dynamic Microfluidic Raman Technology

**DOI:** 10.3390/molecules25071652

**Published:** 2020-04-03

**Authors:** Nga-Tsing Tang, Richard D. Snook, Mick D. Brown, Bryan A. Haines, Andrew Ridley, Peter Gardner, Joanna L. Denbigh

**Affiliations:** 1 Manchester Institute of Biotechnology, University of Manchester, 131 Princess Street, Manchester M1 7DN, UK; k.tang1116@gmail.com (N.-T.T.); Richard.snook@manchester.ac.uk (R.D.S.); 2 School of Chemical Engineering and Analytical Science, University of Manchester, Manchester M13 9PL, UK; 3 Division of Cancer Sciences, University of Manchester, Manchester M20 4GJ, UK; Michael.d.brown@manchester.ac.uk; 4 Fluxion BioSciences, 1600 Harbor Bay Parkway, #150, Alameda, CA 94502, USA; Bryanahaines@gmail.com; 5 Labtech International Ltd., Mytogen House, 11 Browning Road, Heathfield, East Sussex TN21 8DB, UK; ar@cellink.com; 6 Biomedical Research Centre, School of Science, Engineering and Environment, University of Salford, Salford M5 4WT, UK

**Keywords:** Raman spectroscopy, single cell analysis, prostate cancer, fatty acids, microfluidic device

## Abstract

It is known that intake of dietary fatty acid (FA) is strongly correlated with prostate cancer progression but is highly dependent on the type of FAs. High levels of palmitic acid (PA) or arachidonic acid (AA) can stimulate the progression of cancer. In this study, a unique experimental set-up consisting of a Raman microscope, coupled with a commercial shear-flow microfluidic system is used to monitor fatty acid uptake by prostate cancer (PC-3) cells in real-time at the single cell level. Uptake of deuterated PA, deuterated AA, and the omega-3 fatty acids docosahexaenoic acid (DHA) and eicosapentaenoic acid (EPA) were monitored using this new system, while complementary flow cytometry experiments using Nile red staining, were also conducted for the validation of the cellular lipid uptake. Using this novel experimental system, we show that DHA and EPA have inhibitory effects on the uptake of PA and AA by PC-3 cells.

## 1. Introduction

It has been known for many years that metabolic reprogramming, resulting in increased production of intermediates for the synthesis of proteins, nucleic acids and lipids, is a well-established signature of cancer development [1,2]. This process is a prerequisite for cells to break-free from the primary tumour, survive in an energy/nutrient deficient environment, and migrate to a metastatic niche [3,4,5,6]. Within the last decade or so, it has become increasingly clear that the function of cellular lipids is not just energy storage, but these biomolecules play a crucial role in cellular regulation associated with cancer progression. Cells in healthy mammalian tissues satisfy their lipid requirements through the uptake of fatty acids (FAs) from the bloodstream and this is an important and tightly controlled process [7]. Metabolic reprogramming initiates a shift in the balance between exogenous FA uptake and de novo FA synthesis. The deregulation of FA uptake in cancer metabolism and consequences for cancer progression; however, are still not well understood [8,9]. 

FA metabolism is a complex and dynamic process and there is a real need to develop new analytical methodologies that enable the study of such systems in real time under changing dynamic flow conditions. Uptake studies have been carried out using a range of biological assays including fluorescent imaging [10]. Fluorescent labels can either be general or specific for a given FA, but the use of such labels is not always straightforward since the label itself can affect the uptake and biochemical activity of the labelled molecule [11]. Detailed chemical information can potentially be gained from mass spectrometry (MS)-based studies but typical lipidomic experiments involve both extraction of lipids from the biological sample and chromatographic separation prior to analysis [12]. Importantly, MS-based lipidomic experiments cannot be done at the single cell level and although single cell lipid imaging using time of flight secondary ion mass spectrometry (ToF-SIMS) can be achieved, it is not high throughput and generally only images from a few cells are reported [13]. More recently, studies have been carried out using optical techniques such as infrared (IR) or Raman spectroscopy. Unlike MS, these methods are non-destructive and can measure the overall global biochemistry of a sample on a cell by cell basis. FA uptake studies have mostly sampled fixed time points under static rather than dynamic flow conditions [14,15,16,17]. Infrared studies by Gazi et al. followed the uptake of both deuterated arachidonic and palmitic acid but the samples were analysed after being fixed and dried since strong IR absorption by water significantly hinders live cell analysis [14]. Raman spectroscopy is more suited to the study of cells in aqueous environments since the water gives a relatively weak Raman spectrum. Lipid molecules modified with an alkyne tag have been employed to the study specific lipid uptake [17,18,19]. The alkyne tag can show a much higher Raman signal intensity in the spectra and hence enables easier monitoring of the uptake of the tagged molecule. The uptake and metabolism of fatty acid molecules, however, are highly sensitive to structure changes, which raises a question of whether the tagging could affect the metabolic pathway [20]. 

To study FA uptake under more realistic conditions it is desirable to couple spectroscopy with a microfluidic device such that real time measurements under dynamic/switchable conditions can be measured. Coupling Raman to a dynamic flow system is a relatively new development and has been used to investigate several biological systems including identification of circulating tumour cells, drug cell interaction and lipid uptake [21,22,23,24,25]. Most studies use surface enhanced Raman spectroscopy (SERS) to increase the sensitivity of the system but again the addition of the SERS tag to the molecule in question has the potential to influence the outcome. Often these studies use home-made bespoke microfluidic devices that are tailored specifically to a given task and are thus not readily available [21,22,23,24,25]. In the work presented here, a commercially available shear-flow assay system (BioFlux200, Fluxion Biosciences Inc., Ca, USA) is used to provide a dynamic flow environment coupled to a Raman microspectrometer. This represents the first demonstration of this particular combination of instrumentation. In this study, we use the prostate cancer cell line PC-3 to monitor the uptake of various fatty acids.

Prostate cancer (PCa) is the third most common cancer in the UK and the second most common cause of cancer death among men [26]. Metastases to the bone marrow stroma (BMS), a rich source of lipids stored within adipocytes, is the main cause of morbidity and mortality in PCa patients. Several studies have demonstrated how dietary fatty acids relate to the progression of PCa and in recent years, it has been shown that specific types of FAs have a particularly detrimental effect on patient outcome [27,28,29]. Moreover, research has shown a strong correlation between different types of dietary fatty acids (FAs) intake and PCa progression, where high-levels of palmitic acid (PA) and arachidonic acid (AA) intake tend to have a greater impact on cancer behaviour [27,28,29]. Brown et al., however, found that omega-3 polyunsaturated fatty acids (PUFAs) can induce inhibitory effects on the uptake of AA [30]. This finding has implications for competitive lipid uptake between different types FAs. In the work reported here, isotopically labelled FAs (deuterated palmitic acid (D31-PA) and deuterated arachidonic acid (D8-AA)) were used in combination with two non-deuterated omega-3 PUFAs, docosahexaenoic acid (DHA) and eicosapentaenoic acid (EPA) to study competitive uptake in PC-3 cells.

## 2. Results

### 2.1. Example Cell Spectra

**Figure 1:**
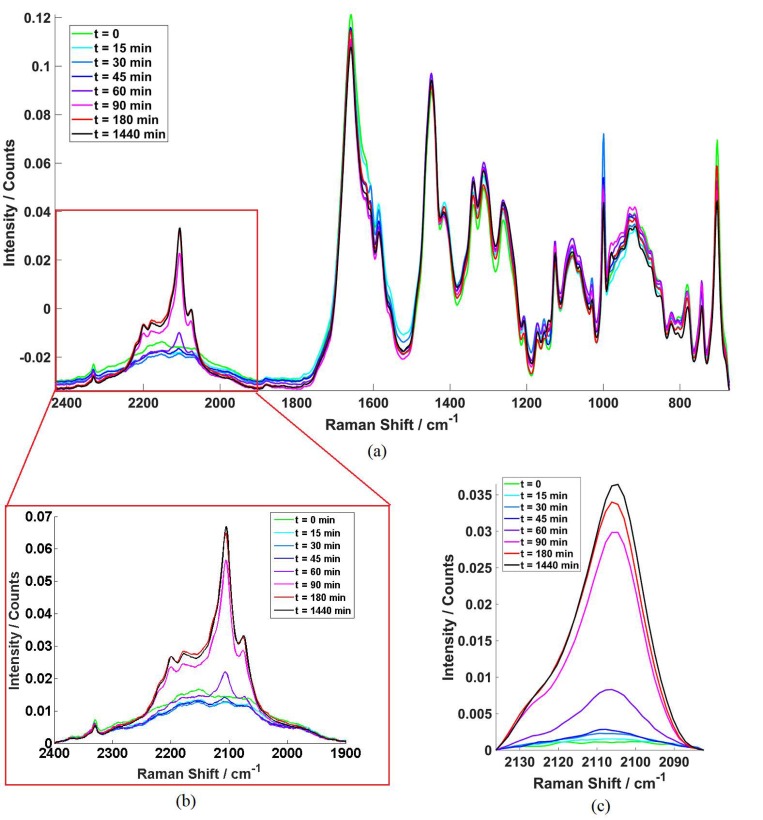
Example mean cell spectra at each time point (PC-3 Cells treated with D31-PA under normoxic condition) with different Raman shift ranges. (**a**) Full range mean cell spectrum. This contains both the cell information and the CD signal acquired in the ‘silent region’. (**b**) Mean spectra in the CD stretching region (1900–2400 cm^−1^) zoomed in from (**a**). This contains the whole CD stretch spectral envelope from the D31-PA. (**c**) Mean spectra of the 2106 cm^−1^ CD peak used (2082–2136 cm^−1^). This is the Raman shift region that was used for relative quantification of the D31-PA uptake by the PC-3 cells by calculating the areas under the curve in this study.

Raman spectra of PC3 cells exposed to D31-PA were acquired from 671 to 2430 cm^−1^ within which 1900-2400 cm^−1^ is the CD stretching region for monitoring the uptake of the deuterated FAs [31]. During the measurement, each single cell was targeted individually with the Raman beam. Example microscope image of cells can be found in the Section Appendix A.1. Figure 1a,b shows examples of all the mean spectra at each time point over full spectrum range and CD stretching region respectively for the cells treated with D31-PA only under normoxic condition (oxygen concentration in a normoxic incubator is 18.6%, at sea level [32]). Figure 1c shows the mean spectra of the 2106 cm^−1^ peak (2082–2136 cm^−1^) used for monitoring the D31-PA uptake.

As shown in Figure 1b, the baseline of the CD stretching region is not flat which means the background signal was not able to be removed completely. Nevertheless, the symmetric –CD_2_ stretching (*ν*_s_CD_2_) signal from D31-PA can be clearly detected at 2106 cm^−1^. Figure 1c shows just the region 2082-2136 cm^−1^ with a linear baseline correction. The areas under these curves were subsequently used for monitoring the relative, rather than absolute, D31-PA uptake. 

### 2.2. Competative Fatty Acid Uptake.

**Figure 2:**
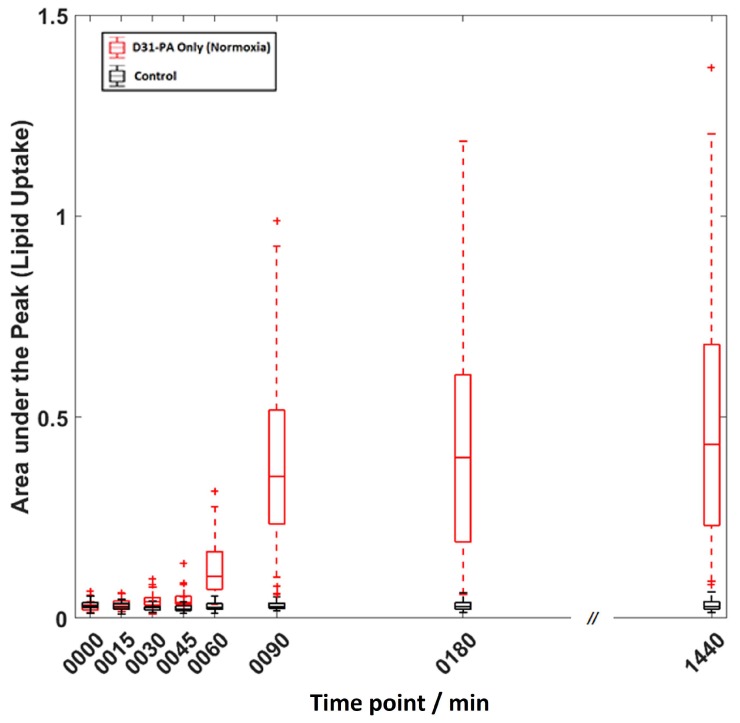
Example box-and-whisker plot for cells treated with D31-PA only under normoxic state and the control experiment. On each box, the central line is the median while the edges of the box are the 25th and 75th percentiles. The whiskers extend to the most extreme data points that are not outliers (within ~99.3% of the data boundary), and the outliers are plotted individually as cross marks.

Areas under the *ν*_s_CD_2_ peak were calculated for each cell to monitor the relative D31-PA competitive uptake. D31-PA is the only isotopically tagged material used in the treatment and all Raman spectra were acquired from cells in pure DPBS. Therefore, the *ν*_s_CD_2_ peak at 2106 cm^−1^ indicates only D31-PA uptake by the cells. Figure 2 shows and example box and whisker plot for cells treated with D31-PA under normoxic conditions. The trend plot shows that there is a continuous increase in the D31-PA uptake as a function of exposure. There is, however, significant variation in the amount of D31-PA taken up by individual cells as a function of time, indicative of cell population heterogeneity. 

**Figure 3:**
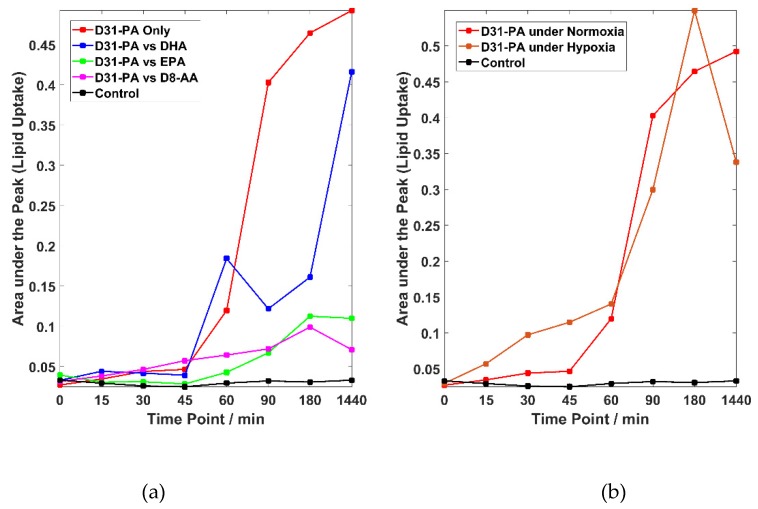
Lipid uptake experiments under both normoxic and hypoxic conditions. (**a**) Trend lines linking the means for the D31-PA uptake by cells for the experiments carried out under normoxic conditions from *t* = 0 to *t* = 1440 min. (**b**) Trend lines linking the means of the D31-PA uptake by cells treated with D31-PA alone under normoxic and hypoxic states from *t* = 0 to *t* = 1440 min. Please note that (i) for display purposes there is a discontinuity of the time axes between 180 and 1440 min and (ii) the trend lines in (a,b) are only a guide for the eye.

Figure 3a shows competitive fatty acid uptake under normoxic conditions. Please note that the heterogeneity in uptake is similar to that observed in Figure 2, but for visual clarity just the trend lines are shown. The cells treated with D31-PA alone have the highest uptake rates (the red line). The trend line suggests that the uptake of D31-PA started as soon as the treatment was administered and a notable increase of D31-PA signal can be seen 45 min post treatment. This uptake remains at a high rate but starts to slow down after 90 min. After 24 h of treatment, the uptake of D31-PA was still on-going but at a slower rate. 

Lipid competition experiments were carried out by treating the cells with D31-PA and one other FA: DHA, EPA or D8-AA under normoxic conditions. The trend lines of the lipid competition experiments are also plotted in Figure 3a where suppression of the uptake of D31-PA by the PC-3 cells due to the presences of DHA, EPA or D8-AA can be seen to varying degrees. Since D31-PA is the only reagent used that will give signal at the 2106 cm^−1^ *ν*_s_CD_2_ peak, inhibitory effects on the D31-PA uptake by omega-3 FAs can be reflected if a lower rate of uptake of D31-PA is observed. There are no significant uptake differences between all experiments in the first 45 min of treatment, yet suppression of the D31-PA uptakes by DHA, EPA and D8-AA were observed from *t* = 60 min. This suppression effect can be seen throughout the rest of the time points in the EPA and D8-AA experiments. However, cells treated with D31-PA and DHA behave differently compared to the other experiments. Although it was expected that DHA will have the similar suppression effects on the uptake of D31-PA by the PC-3 cells, the results suggest DHA reduces uptake but it is not as effective as similar concentrations of AA or EPA. 

Data show the suppression of D31-PA uptake in the presence of D8-AA under normoxic conditions, suggestive of the essential nature of AA in contrast to the non-essential PA. In Figure 3a, however, the uptake trend of D31-PA by the cells treated with D31-PA only is slightly different to other cases. The uptake of D31-PA maximises at *t* = 180 min and starts decreasing afterwards in the lipid competition experiment with D8-AA, where the uptake of D31-PA gradually increases after *t* = 180 min in other cases. It is believed this phenomenon is related to the cellular adaptation to the environment. 

The coupled system used in this study can also be used for studying similar mechanisms under different conditions. To demonstrate this flexibility, uptake of D31-PA by PC-3 cells was also monitored under chemically induced hypoxic conditions. The majority of the research into the relationship between the progression of PCa and dietary FAs are carried out under the “normoxic” atmospheric conditions. However, it has been reported that many prostate tumour lesion are hypoxic, with an average pO_2_ of 6.7 mm Hg as compared with blood (75–100 mm Hg) and muscle (30 mm Hg) [33]. These changes in conditions may affect the metabolism of cells but not necessarily make any difference to the uptake of lipids. The trends for the uptake of D31-PA under normoxic and hypoxic conditions are compared in Figure 3b and the result suggests that the uptake of D31-PA by PC-3 cells under hypoxic condition started earlier than cells under normoxic conditions. D31-PA signal can be detected at 15 min of treatment and continues to increase until reaching a maximum at 180 min. The trend of the uptake of D31-PA under hypoxia drops at 24 h. 

All results have been statistically tested by comparing the D31-PA uptake by cells treated under normoxic conditions with the competition experiments using the two-tailed t-test. The relative information and statistical results can be found in Table 1. The null hypothesis is that the uptake of D31-PA by PC-3 cells stays the same under different conditions. The *p* value for each case was calculated and any *p* value that is greater than the critical value of 0.05 or 0.01 will reject the null hypotheses, i.e., there are significant differences between the two sets of data. Table 1 shows the calculated *p* values for all cases at each time point. The results show that the uptake of D31-PA is supressed when omega-3 fatty acids were administered to the cells along with the D31-PA. Furthermore, a significant difference of D31-PA uptake between the control experiment and the cells treated with D31-PA only was observed.

Further Student’s *t*-tests were performed on the data to confirm that there are statistical differences of the D31-PA *ν*_s_CD_2_ peak area, between the experiments using untreated and treated cells. The table showing the *p* values and further information can be found in Section Appendix A.3. 

### 2.3. Distribution of Data Points

**Figure A2:**
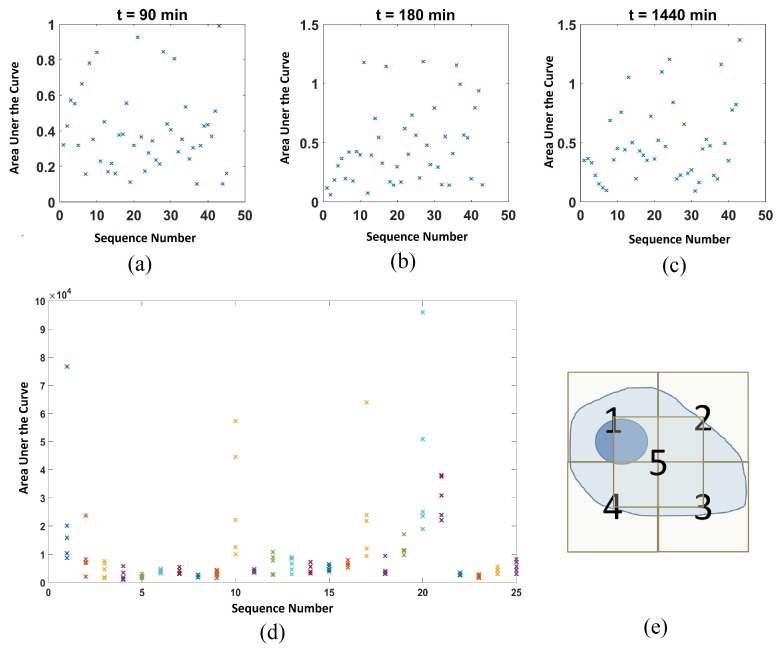
Distribution of the amount of lipid taken up by cells. Top row showing the example single point results of area under the curve against corresponding event number for cells treated with D31-PA only under normaoxic conditions, at time point (**a**) 90 min, (**b**) 180 min, and (**c**) 1440 min. (**d**) Representative plot for the area under the 2106 cm^−1^ peak at the five measurement locations in each cell at *t* = 60 min. (**c**) Corresponding schematic of the locations of the five measurements taken in the experiment.

It was observed that there are larger variances in D31-PA uptake between cells at later time points. This is unlikely to be caused by the system’s stability, as the system had been tested non-biologically with polystyrene beads (details can be found in Section 4.1.3). To rule out a relationship between the variance in measured uptake and the position in the sequence of 45 cell measurements, the relationship between the areas under the *ν*_s_CD_2_ peak and the corresponding sequence number was investigated for three time points. As can be seen in Figure A2a–c, no trend exists between the level of uptake and the order in which the cells were measured 

A more likely cause of variation in the data is the localisation of lipid droplets within the cells [34,35]. The Raman laser spot size is smaller than the whole cell, so it is possible that large differences will be seen if the Raman laser hits a lipid droplet rather than an area with low lipid concentration. To investigate this, cells were treated with 20 µM D31-PA but in this case five spectra were obtained for each cell at different locations, which means that the whole cell was completely sampled, see Figure A2d,e. 

The areas under the peaks measured at the five locations in each cell are different, reflecting the localisation of the lipid droplets. This may be one of the contributions to the large variance observed at later time points in the lipid uptake experiments, where the Raman laser might have just hit or missed the lipid droplet. In addition to the variations within each cell, variations between different cells were also observed.

## 3. Discussion

In this study, the BioFlux microfluidic system was coupled with a Raman microspectrometer for the first time, and therefore several system optimisation and method development experiments were required. Cells had to be seeded into the microfluidic device before performing any experiments. Coatings, such as poly L-lysine or fibronectin, are widely employed for improved cell attachment in microfluidic channels [36,37]. Although the coating is only a monolayer of molecules, it may still be detected by Raman spectroscopy, which is undesired. Therefore, it was decided to pre-seed the cells for 2 days in uncoated wells before starving so that the cells had enough time to settle down and attach. Cells seeded with this method allowed us to perform the experiments with no significant cell detachment at a sheer flow rate of 0.15 dyn cm^−2^.

To ensure a consistent FA baseline, serum-free media was used in the FA uptake experiments. This is because serum-containing culture media will lead to a reduction in the D31-PA uptake compared with cells cultured in serum-free medium, specifically due to the presence of foetal calf serum (FCS) which contains a range of FAs [38]. A FA treatment concentration of 20 µM was selected based on the study by Brown et al., which suggested that cell viability decreases in the presence of FA at a concentration greater than 50 µM [30]. In the work present here, a total FA concentration of either 20 µM (single FA), or 40 µM (competitive uptake) was employed.

One of the aims of this study was to monitor a specific FA uptake which normally would require a tag on the FA. However this may affect the uptake and metabolism of the FA as FAs are highly sensitive to structure changes [20]. Deuteration overcomes this problem and has the added benefit of the resultant Raman signal of CD stretches appearing in the region of 2000–2400 cm^−1^ where no other biochemical information is observed. Furthermore, deuteration has minimum influence on the metabolic pathway of the FAs. D31-PA gives the strongest signal at 2106 cm^−1^ which is attributed to the *ν*_s_CD_2_. D8-AA was also used in one of the experiments and the = CD stretch was expected to show at 2250 cm^−1^. This is because D8-AA was deuterated at the unsaturated carbons in the molecule, where D31-PA was deuterated at the saturated carbons. This results in the increase in vibration frequency of the CD stretches in the D8-AA compared with the D31-PA [39]. However, no D8-AA signal was detected in the Raman spectra acquired in this study. This is suspected to be due to the low Raman sensitivity of, *ν*(= CD), compared to the *ν*_s_CD_2_ signal [40]. Since these two peaks are very close to each other and a flat baseline was not achieved in the CD stretching region, the weak *ν*(= CD) signal from D8-AA appears to have been obscured by the dominant *ν*_s_CD_2_. 

Brown et al. demonstrated that omega-3 FAs have an inhibitory effect on the invasive ability of PC-3 cells induced by AA. The invasive effect could be recovered again by the addition of downstream AA metabolites [30,41]. However, it was not conclusive that the inhibition was due to metabolic competition or competitive uptake. With PA, also a FA that induces metastatic effects of PC-3 cells [42,43], it is hypothesised that the uptake could be inhibited by omega-3 FAs such as DHA and EPA. The Raman results acquired here suggest that the omega-3 FAs do inhibit the uptake of PA by the PC-3 cells. However, the uptake of PA by the cells treated with both D31-PA and DHA shows a different trend to the other cases. The suppression of the D31-PA uptake is much lower, and the trend line suggests an increase in uptake after 24 h (1440 min) of treatment. This suggests the inhibitory effect of the D31-PA uptake by DHA is less effective than EPA or AA, which is consistent with the invasion findings by Brown et al. [30]. Similar inhibitory effects on the absorption of saturated FAs, such as PA, brought about by EPA and DHA have also been observed by Yang et al. [44]. 

From the results presented here, D31-PA can be clearly detected using Raman microspectroscopy and trends in D31-PA uptake can be obtained under different environmental conditions. However, large variations in the *ν*_s_CD_2_ signal detected were found in the live-cell experiments, especially in the later time points. Relative standard deviations (RSDs) were calculated from the cell spectra acquired. Despite the RSD associated with D31-PA uptake at time points 90, 180 and 1440 min being 60–70%, the RSD for the whole spectrum range is only ~4.5%. (The corresponding tables can be found in the Section Appendix A.3.) System repeatability using polystyrene beads was determined to be greater than 99% (Section 4.1.3), and therefore it is believed the variations observed in uptake are purely because of the biological variances between single cells. This can be further demonstrated by the results obtained from the experiments shown in the Section Appendix A.2, in which the entire cell was measured. Although variations of D31-PA signal were observed due to lipid localisation, there is also significant variation of the CD signal between cells. 

The uptake of D31-PA displayed in Figure 3 generally shows a decrease after treatment of 180 min, which is believed to be attributed to the adaption of the cell to the environment as discussed in Section 2.2. The cells were starved for 24 h in serum-free media before performing the uptake experiment; thus depriving the cells of lipids required for survival and proliferation and ensuring a comparable lipid baseline for all the experiments. PA can be synthesised by the cell from carbohydrates or protein (available resources from the media) while AA can only be obtained from diet [45], cells will only uptake the essential FAs (AA in this case) after adapting to the environment. 

PA uptake by PC-3 cells under chemically induced hypoxic conditions was also studied. Some differences in the uptake of D31-PA were observed at 30 min and at 24 h with an increase of hypoxic uptake at 30 min but a decrease after 24 h. Bensaad et al. reported that hypoxia can induce greater uptake of lipids from the extracellular environment where there is an increase in lipid droplets upon HIF1-α induction without an increase in intracellular production [46]. However, this needs further investigation.

## 4. Materials and Methods 

### 4.1. Instrumentation

**Figure 4:**
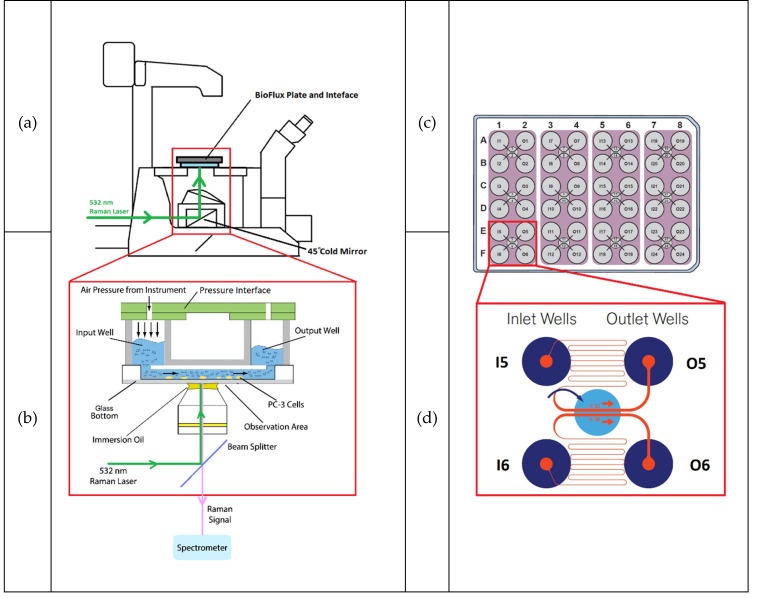
Schematic diagram of the system layout and equipment configuration. (**a**) Side view of the configuration of the microscope and the microfluidic plate with the plate connecting to the main body of the BioFlux system. Green arrow showing the inlet Raman laser path to the sample stage. The area in the red rectangle is expanded in (**b**), which shows the configuration of the measuring unit (BioFlux interface, the well plate, the microscope objective and a simplified light path diagram). (**c**) Top-down view of the BioFlux 48-well plate configuration. (**d**) Layout of the microfluidic channels (one observation unit, showing two pairs of channels, only one pair is used at a time). In the top pair the media and FA would flow from the inlet well (designated I5) through the hairpin microfluidic channel to the observation window containing the cells, then back out to the outlet well (designated O5). The Raman beam would be focused on the cells in the top channel in the observation window.

In this study, the BioFlux 200 shear flow assay system (Fluxion Biosciences Inc., Ca, USA) was coupled with a customosed Raman microspectrometer and several optimisation and method development experiments were carried out. The final system configuration is shown in Figure 4. Figure 4a,b shows the layout of the coupled BioFlux and Raman microspectrometer and 4c,d shows the configurations of the 48-well plates used in this study. 

The BioFlux 200 contains a controller, a pressure interface, a vapour trap, pipelines for connecting the units, and a heating plate. The controller regulates the air pressure applied to the wells and also the heating plate temperature. Experiments were conducted in the BioFlux well plates coupled to a Raman microspectrometer, which consists of an inverted Nikon Eclipse TE300 microscope (equipped with a Nikon Plan Fluor 100 × oil immersion objective, Nikon UK Ltd., Surbiton, UK), a Horiba Scientific iHR-320 Raman Imaging Spectrometer (focal length of 320 mm, f/4.1 aperture), coupled with a Horiba Syncerity CCD camera (Horiba Scientific, Northampton, UK). A beam splitter (Edmund Optics NT64-286), was used for guiding the 532 nm Raman laser to the sample stage and also separating the reflected excitation laser and the Raman signal going to the spectrometer. 

#### 4.1.1. Microfluidic Device

The BioFlux well plates required for the system are the size of standard 24- or 48-well plates, but with microfluidic channels built underneath the wells. The main body of the well plates are made of polystyrene, while the top and the walls of the microfluidic channels are made of polydimethylsiloxane (PDMS). Since a high numerical aperture is used for the Raman spectroscopy, the laser beam shape is no longer collimated. Thus, there is a chance of getting some PDMS signal scattered from the top and walls of the channel. Although PDMS is highly Raman sensitive and could potentially interfere with the experiment, this is not a problem since the CD signals from the deuterated lipids are located in the ‘silent area’ of a Raman spectrum in which PDMS does not contribute. The dynamic flow process is controlled through the BioFlux software, for which manual and automatic modes are available. The air pressure can be set from 0 to 20 dyn cm^−2^ and the length of operating time can be pre-set for the automatic mode. In Figure 4c, the purple shading on the well plate indicates one operation unit, with inlet at the left hand side and outlet at the right hand side. The air pressure can be used to control flow rate and can be applied on either the inlet or outlet side in each unit and the four units can be operated at the same time. Moreover, due to the pump design in the main controller, the air pressure applied has to be the same for each of the two operation units (Column 1–4 and Column 5–8). However, length of operation time can be varied. 

A sheer flow of 0.15 dyn cm^−2^ was chosen for the experiment, which is high enough to enable a continuous supply of both fresh media and FAs to the cells in the microfluidic channels but is not high enough to cause detachment of cells from the channel surface.

A capillary structure consisting of a series of bends allows laminar flow to be developed in the microfluidic channels as shown in Figure 4b. The solution takes a certain length of time to flow from the inlet well to the viewing chamber (dead volume), and the time required will vary depending on the flow rate of the solution. To determine this time, the Raman signal changes were monitored as RPMI 1640 cell culture medium was replaced by Dulbecco’s Phosphate-Buffered Saline (DPBS, Sigma-Aldrich Inc., Poole, UK). DPBS is a physiological buffer (pH 7.1–7.5 Osmolality 275–304 mOs Kg^−1^) that gives negligible fluorescence background for Raman measurements. Due to the large background intensity differences between RPMI 1640 cell culture medium and DPBS, the total area under the Raman curves (AUC) can be used as an indicator for determining the solution flowing in the microfluidic channels. The results used to obtain the dead time are shown in the Section Appendix A.4. 

#### 4.1.2. Raman Microspectroscopy

To minimise the potential photodamage to the cells, it is important to consider the length of time the cells are exposed to the laser. To obtain a reasonable signal to noise ratio (SNR), the maximum power available from the 532 nm Raman laser at 110 mW was chosen. However, the laser power diminishes to 41.5% of the power at source when it reaches the sample stage on the microscope. To avoid compromising SNR and signal saturation, several integration times have been tested for acquiring cell spectra at 110 mW at laser source. The final integration time used for all cell spectra was 10 s. A plot of measured power density and laser output power can be found in the Section Appendix A.5. Please note that all measurements were carried out in a DPBS background, i.e., cells in DPBS. The corresponding cell viability test results can be found in the Section Appendix A.6. With the integration time of 10 s, the time required for acquiring one set of data at a time point was 15–20 min. The test results suggest that cell viability remains greater than 90% after 60 min, which is longer than the time required for all Raman spectral acquisition at each time point. After the measurement, cells were then exposed to culture medium again to provide materials essential for their survival. 

#### 4.1.3. System Repeatability

The repeatability of the system was tested using polystyrene beads. All measurements were taken with Raman power of 55 mW at laser source (to avoid detector saturation) and 1 s integration time. Two sets of experiments were conducted to test the repeatability of the system with 20 μm and 25 μm diameter polystyrene beads:

(i) Repeat measurements on the same bead

45 measurements on the same polystyrene bead were acquired sequentially. Two more replicates were obtained by moving the laser beam away from the bead for a few minutes, then returning to the same bead again for another 45 measurements. This experiment was repeated for three times i.e., three replicates.

(ii) Repeat measurements on different beads

45 different beads were measured sequentially. Three replicates were taken, i.e., the experiment was repeated three times, but beads measured are not necessarily the same beads among the three replicates.

**Figure 5:**
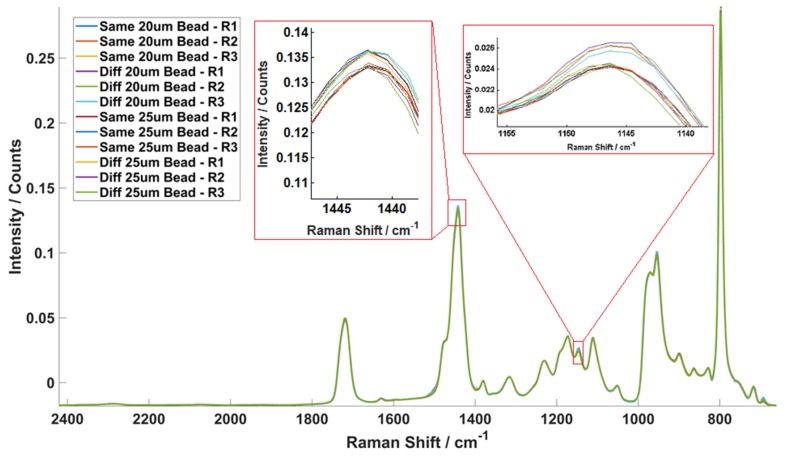
System repeatability test results and the corresponding statistical test results showing the mean spectra of all 12 polystyrene bead experiments. All 12 mean spectra generated from 540 single bead spectra overlay with each other in the figure, showing that there are negligible differences between the Raman spectra measured in different situations. The calculated relative standard deviations of each case and the calculated average RSD is only 0.94%. The inserts show the peaks at 1442 and 1145 cm^−1^ in order to better highlight the individual spectra.

Figure 5 plots all the mean spectra of the three replicates of the experiments. In each case, there were 45 measurements and therefore there are 540 measurements in total. As can be seen there is almost perfect overlap between spectra. To observe marginal spectral differences, two sections have been significantly expanded and are shown in the insert in Figure 5 Standard deviations (SD) of the AUCs, mean AUC and relative standard deviations of the AUCs were then calculated. An average RSD of 0.94% was obtained from all 12 experiments indicating that the Raman microspectrometer system produces stable performance and gives high repeatability (>99%). This confirms that variances obtained in experiments involving cells are caused by biological differences. 

### 4.2. Cell Culture

The PC-3 cells were first cultured in standard culturing flasks in Ham’s F12 cell culture medium (Sigma-Aldrich Inc.), 10% foetal bovine serum (FBS, Sigma-Aldrich Inc.), 1% L-glutamine (Sigma-Aldrich Inc.) and 1% penicillin/streptomycin (Sigma-Aldrich Inc.) until 70% confluency. Cells were pre-seeded into the channels at a concentration of 2 × 10^7^ cells mL^−1^ two days prior to the experiment to allow adhesion before treatment. Cells were seeded from the outlet side of the channel to prevent any contamination of the inlet channel at a sheer flow of 2 dyn cm^−2^ for 5 s. During these primary cell culture times, the cells were incubated at 37 °C with 5% CO_2_ supply in air.

Before applying any treatment to the cells under normoxic conditions (oxygen concentration in a normoxic incubator is 18.6%, at sea level [32]) in microfluidic channels, the cells were starved in serum-free RPMI 1640 medium (Sigma-Aldrich Inc.), 1% L-glutamine and 1% penicillin/streptomycin for 24 h. During the starvation period, the cells were also incubated at 37 °C with 5% CO_2_ supply in air. 

In this work hypoxia was chemically induced with cobalt (II) chloride (CoCl_2_, Sigma-Aldrich Inc., Poole, UK) dissolved in distilled water [47]. 100 μM of CoCl_2_ was added to the serum-free medium and the cells were starved and incubated with the CoCl_2_ for 24 h before the lipid uptake experiment. The induction of hypoxia in these cells was validated using Image-iT™ Red Hypoxia Reagent (Thermo Fisher Scientific UK Ltd. Hemel Hempstead, UK), a reversible fluorescent dye for measuring hypoxia in live cells. This reagent works based on the intracellular oxygen level which is non-fluorescent when the intracellular oxygen level is above 5% and becomes fluorescent (in red) when the intracellular oxygen level is below 5%. The results of these experiments are described in the Section Appendix A.7.

### 4.3. Fatty Acid Treatments 

Several treatments were applied in different experiments to study the competition between the D31-PA with other fatty acids (FA). All FAs used in this project were obtained in either powder or neat oil form. Ethanol (≥ 98%, Thermo Fisher Scientific UK Ltd. Hemel Hempstead, UK) was chosen as the vehicle to make the FAs available to the cells. The concentrations used for each FA was 20 μM in serum-free RPMI 1640 cell culture medium with 1% L-glutamine and 1% penicillin/streptomycin. The cells were treated with either: D31-PA only, D31-PA and DHA, D31-PA and EPA, and D31-PA and D8-AA in each experiment (all FAs are from Sigma-Aldrich Inc., Poole, UK). 

The FA-containing RPMI 1640 cell culture medium (with 1% L-glutamine and 1% penicillin/streptomycin for all experiments, and 100 μM CoCl_2_ for hypoxia experiment) is supplied to the cells by constantly applying 0.15 dyn cm^−2^ sheer flow to the inlet during the experiment, except during the Raman spectra measurements. At each time point, the solution was then switched to DPBS at the same flow rate until the viewing chamber was fully replaced with DPBS. After the measurement, the solution was then switched back to the FA-containing medium for further treatment. Approximately 45 cells were sampled at each time point: *t* = 0, 15, 30, 45, 60, 90, 180 and 1440 min. 

### 4.4. Data Processing and Analysis

One-to-one background subtraction was applied to each cell spectrum (SynerJY version 3.5.7.20). 

All the cell spectra were imported into MATLAB 2017a for further processing including removal of any contributions from cosmic rays. 3-point smoothing with a moving mean method (chosen to minimise the effect of peak shifts), linear baseline correction and vector normalisation were applied. 

With the 1200 lines per mm grating used in this project, the Raman shift is available from range 671 to 2430 cm^−1^. However, the Raman shift range is purposely reduced to 2082–2136 cm^−1^ for monitoring the *ν*_s_CD_2_ stretch from the D31-PA. The relative uptake of the D31-PA is quantified by calculating the area under the curves in this range after 2-point rubber band correction at first and last point of the spectra. Uptake curves of the experiments were plotted with the mean spectral area under this peak. 

## 5. Conclusions

This work demonstrates the novel combination of coupling the BioFlux microfluidic system with a Raman microspectrometer in order to spectroscopically study biological systems under dynamic flow. With the increase in importance of studying the biology of living cells under their natural environment, development of dynamic flow systems help to simulate physiological conditions. The use of isotopically labelled fatty acids enabled monitoring of the uptake of a specific type of fatty acid by analysis of CD peaks in the Raman spectra. With the combination of this instrumentation and isotopically labelled reagents, continuous chemical monitoring in living cells at the single-cell level can be performed. 

This study also demonstrates analysis of cells under different conditions such as normoxic and hypoxic states. This is a key factor in single cell studies since physiological conditions, particularly in tumours, are often highly hypoxic but most studies are carried out under *in-vitro* normoxic conditions.

## Figures and Tables

**Table 1:** The *p* values of the two-tailed *t*-tests comparing the D31-PA uptake of cells treated with D31-PA-only and other treatments. * is significant at 0.05 confidence level and ** is at 0.01 confidence level. *n* values are shown in the brackets.

*p* Value	D31-PA Only (Normoxic State)							
	0 min	15 min	30 min	45 min	60 min	90 min	180 min	1440 min
vs. DHA	0.999 (98)	0.999 (89)	0.254 (91)	0.023 (95) *	0.999 (89)	<0.001 (87) **	<0.001 (81) **	0.144 (91)
vs. EPA	1.000 (99)	0.043 (94) *	<0.001 (91) **	<0.001 (90) **	<0.001 (93) **	<0.001 (91) **	<0.001 (92) **	<0.001 (91) **
vs. AA	0.987 (96)	0.933 (100)	0.690 (93)	0.964 (98)	<0.001 (93) **	<0.001 (99) **	<0.001 (85) **	<0.001 (93) **
vs. D31-PA (Hypoxic)	0.918 (99)	1.000 (97)	1.000 (90)	1.000 (96)	0.901 (96)	0.016 (94) *	0.871 (92)	0.010 (93) *
vs. control	0.998 (98)	0.012 (93) *	<0.001 (92) **	<0.001 (94) **	<0.001 (95) **	<0.001 (95) **	<0.001 (93) **	<0.001 (91) **

**Figure A1:**
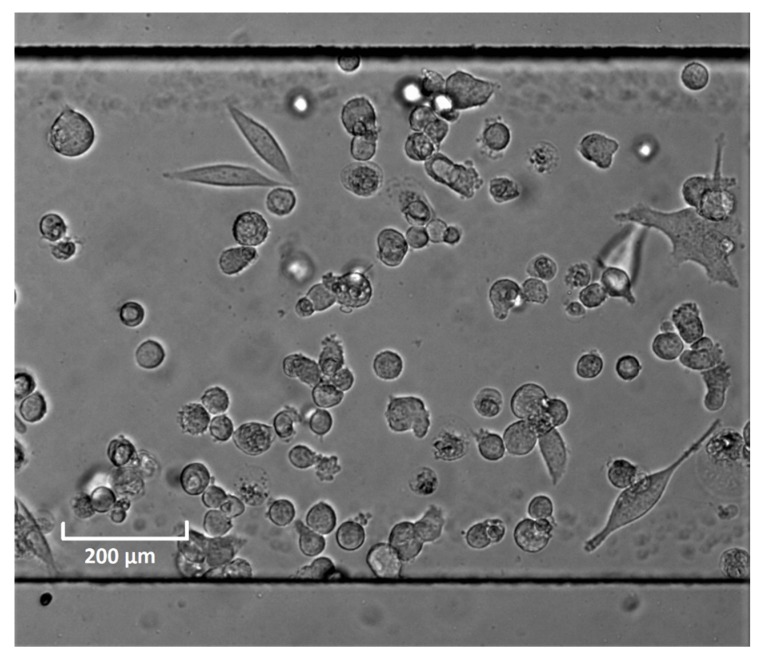
Example image of PC-3 cells cultured inside the microfluidic channel.

**Figure A3:**
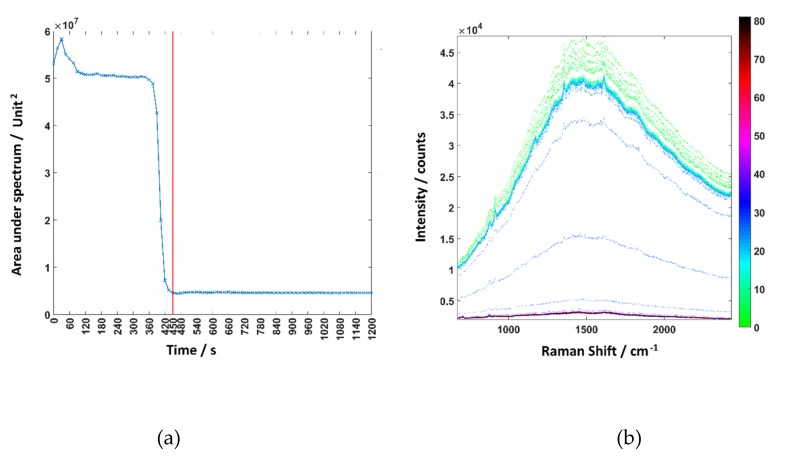
(**a**) The area under the curve of each spectrum in (b) plotted against time. (**b**) Raman spectra of the fluid in the channel acquired at each time point during the flow rate test. The highest curve is the pure RPMI 1640 and the lowest is the pure DPBS.

**Figure A4:**
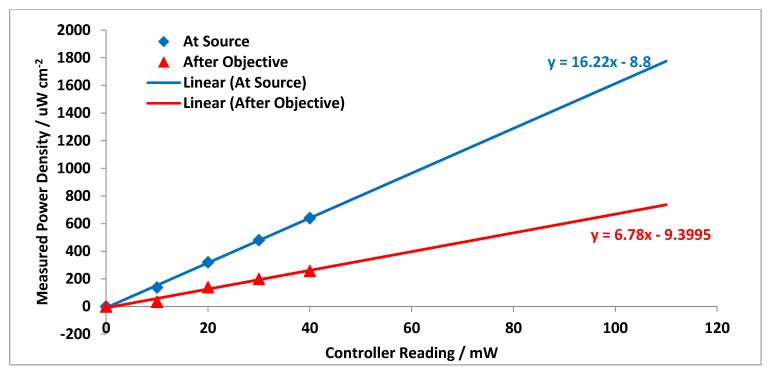
Relative power of the Raman laser at source and at the sample stage.

**Figure A5:**
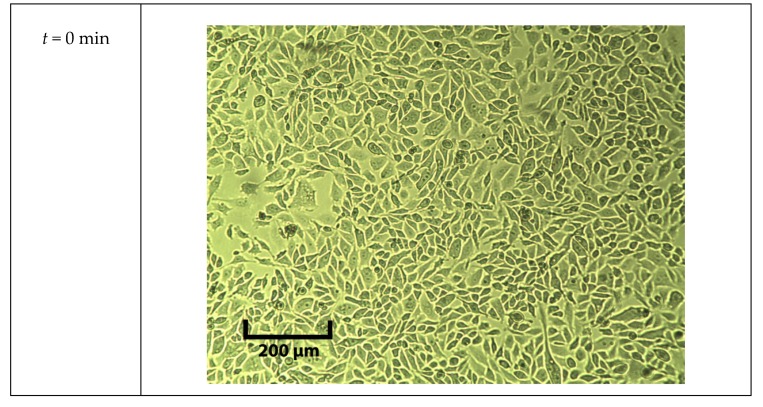

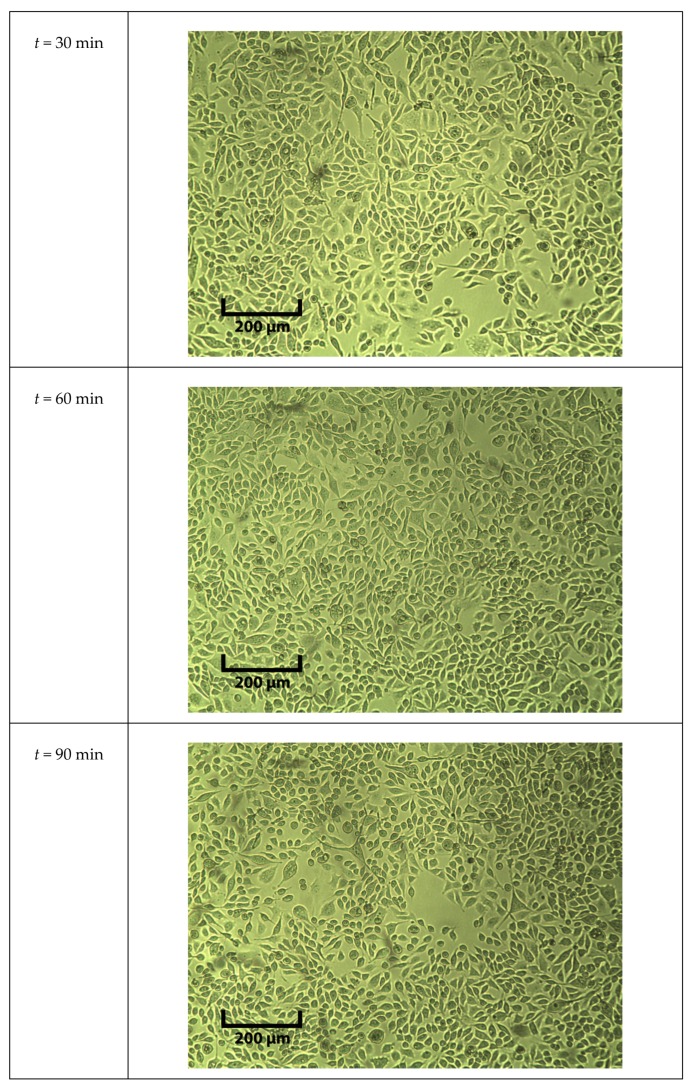
Pictures of cells exposed to room temperature DPBS under 5x objective at different time points.

**Figure A6:**
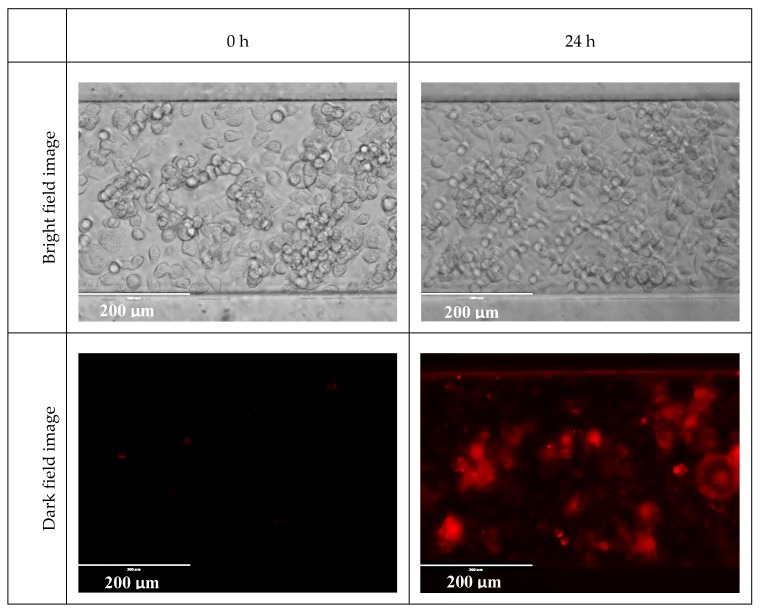
Bright field and dark field microscope images at 0 and 24 h of treatment.

**Figure A7:**
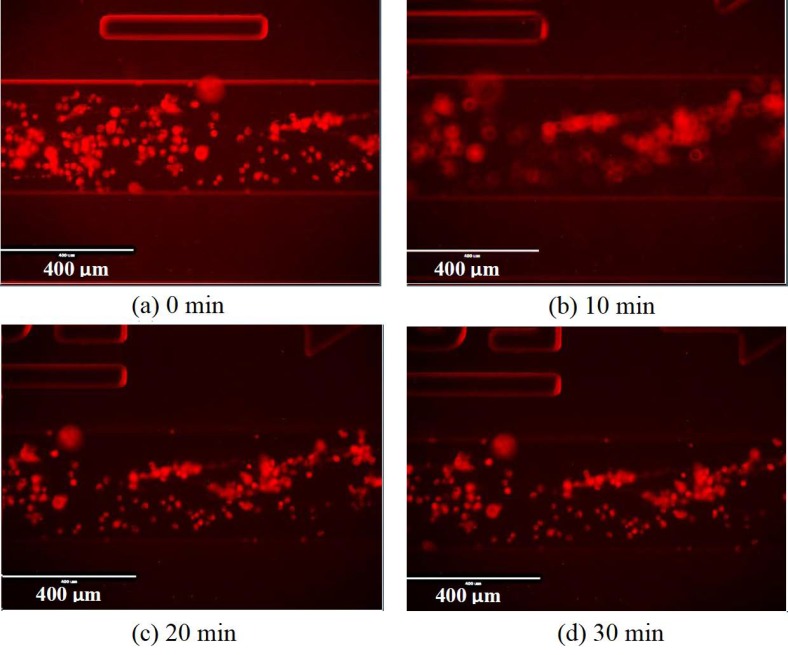
Dark field microscope images for the cells in a hypoxic state exposed to DPBS. (**a**) Image taken at 0 min. (**b**) Image taken at 10 min. (**c**) Image taken at 20 min. (**d**) Image taken at 30 min.

**Figure A8:**
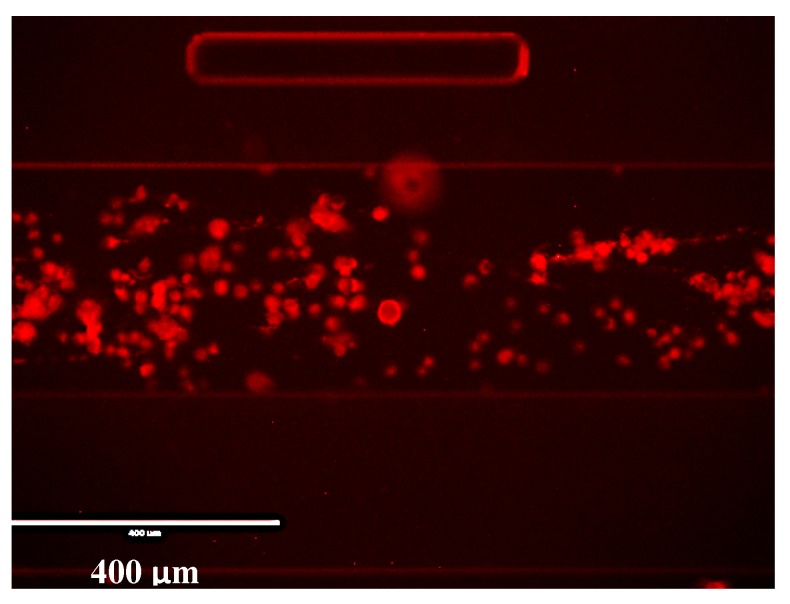
Dark field microscope image of cells exposed to DPBS after 60 min.

## Appendix A

### Appendix A.1. Example Cell Image Taken under the Microscope

Cells under the microscope are shown in Figure A1. During the measurements, the Raman laser was positioned onto the centre of each cell to acquire single cell spectra.

### Appendix A.2. Additional Data on the 5-Point Measurements

As described in Section 4.1.3, the system repeatability was confirmed non-biologically using polystyrene beads and it is believed that the variations observed are purely because of the biological variances between single cells. This was further investigated with ‘five-point measurements’ and the results suggest that the more likely cause of variation in the data is the localisation of lipid droplets within the cells. Results of the ‘five-point measurements’ are shown in, where cells were treated with 20 μM D31-PA. The five measurements at each location ensured that the whole cell was completely sampled.

The areas under the CD peak, plotted against the corresponding sequence number, are shown in Figure A2a–c. A representative plot of the D31-PA signal acquired at each point in a cell at *t* = 60 min is shown in Figure A2d. The locations of the five spectra obtained for each cell are indicated in Figure A2e.

During the experiment, five spectra per cell were taken with 10 s of integration time. Taking the operation time into account, the total laser exposure time per cell was ~1 min. As shown inFigure A2d, the area under the peak measured at the five locations in each cell are different, which reflects the localisation of the lipid droplets. This may be one of the contributions to the large variance observed at later time points in the lipid uptake experiments where the Raman laser might have just hit or missed the lipid droplet. In addition to the variations within each cell, variations between different cells were also observed. From Figure A2b, it is clear that some of the cells give larger *ν*_s_CD_2_ signal than the others indicating that different cells uptake the D31-PA at different rates.

### Appendix A.3. Statistical Significance for the Uptake Experiments

Results obtained were statistically analysed where the relative standard deviations (RSD) were calculated for assessing the variance. The calculated values are shown Table A1 and Table A2 for the area under the 2106 cm^−1^ CD peak and the area under the full spectrum respectively.

**Table A1:** RSD calculated in the D31-PA uptake (2106 cm ^1^ peak only).

	RSD/%							
	0 min	15 min	30 min	45 min	60 min	90 min	180 min	1440 min
D31-PA	32.1	32.5	40.1	46.1	54.1	56.4	69.9	65.2
vs. DHA	27.2	35.2	29.4	31.4	63.3	51.1	56.9	84.9
vs. EPA	40.3	33.3	36.2	29.2	33.8	41.6	54.0	49.3
vs. AA	32.1	34.8	61.9	61.2	86.1	78.1	63.2	51.2
D31-PA (Hypoxic)	32.7	39.1	62.3	64.1	63.0	76.5	69.9	90.9
Ctrl	35.1	34.8	28.5	33.7	35.3	37.8	35.3	44.0

**Table A2:** RSD calculated in the full spectral region.

	RSD/%							
	0 min	15 min	30 min	45 min	60 min	90 min	180 min	1440 min
D31-PA	3.08	2.59	2.73	2.61	2.47	4.08	5.15	4.22
vs. DHA	2.91	2.48	3.08	3.61	3.16	2.56	3.43	4.38
vs. EPA	2.09	1.94	3.55	3.11	3.33	2.94	3.23	2.30
vs. AA	3.21	4.16	2.57	2.51	3.08	3.30	2.87	2.99
D31-PA (Hypoxic)	4.03	3.10	2.46	3.32	3.85	6.02	6.34	11.5
Ctrl	2.91	3.39	3.51	2.46	2.67	3.21	3.41	3.47

Two-tailed *t*-test was also used to evaluate the statistical significance of the uptake of D31-PA by PC-3 cells compared to the control experiment. The null hypothesis is that the uptakes of D31-PA by PC-3 cells are the same between treated and control experiments. The *p* value for each case was calculated and any *p* value that is greater than the critical value will reject the null hypotheses i.e., there are significant differences between the two sets of data. *p* values for all cases are shown in Table A3.

**Table A3:** The *p* values of the two-tailed *t*-tests comparing the uptake of D31-PA in the control experiment with other treatments. * is significant at 0.05 confidence level and ** is at 0.01 confidence level. *n* values are shown in the brackets.

*p* Value	Control							
	0 min	15 min	30 min	45 min	60 min	90 min	180 min	1440 min
vs. D31-PA (Normoxic)	0.998 (98)	0.012 (93) *	<0.001 (92) **	<0.001 (94) **	<0.001 (95) **	<0.001 (95) **	<0.001 (94) **	<0.001 (91) **
vs. DHA	0.543 (98)	<0.001 (88) **	<0.001 (97) **	<0.001 (95) **	<0.001 (90) **	<0.001 (92) **	<0.001 (89) **	<0.001 (96) **
vs. EPA	0.013 (99)	0.272 (93)	<0.001 (97) **	0.025 (90) *	<0.001 (94) **	<0.001 (96) **	<0.001 (100) **	<0.001 (96) **
vs. AA	0.769 (96) *	<0.001 (99) **	<0.001 (99) **	<0.001 (98) **	<0.001 (94) **	<0.001 (104) **	<0.001 (93) **	<0.001 (98) **
vs. D31-PA (Hypoxic)	0.942 (99)	<0.001 (96) **	<0.001 (96) **	<0.001 (96) **	<0.001 (97) **	<0.001 (99) **	<0.001 (100) **	<0.001 (98) **

### Appendix A.4. Biological Optimisation of the System: Flow Rate and Background Test

The intensity differences between the Dulbecco’s Phosphate-Buffered Saline (DPBS) and the RPMI 1640 media are shown in Table A3a.

Figure A3a plots the area under each spectrum shown in Figure A3b against time and shows that the actual time required for replacing one solution in the channel with other solution is 105 s. The time-limiting step of the whole process is to remove the dead volume in the capillary structure. The area under the curve flattens out and remains constant at 450 s in Figure A3a, which indicates the media filled channel is fully replaced with DPBS at this point. This was taken into account when performing the time point experiments.

### Appendix A.5. Relative Raman Laser Power Ratio Measurement Results

To obtain a measure of how much power loss occurs between the laser source and the objective, the relative power was measured at the two locations using an EG & G 550-1 Radiometer/Photometer equipped with an EG & G 550-2 multiprobe detector. For health and safety reasons and in order to make sure that the detector was operating in the linear range, an optical density (OD) 2.0 filter was placed in front of the laser source for the power density measurement. Please note that, since the area of the detector is significantly larger than the laser beam, it is just the relative power between the two locations that is of significance. Data were recorded up to a laser source setting of 40 mW as shown in Figure A4. Extrapolating this data to 110 mW we obtain a power at the objective of ~45 mW. Although this is quite high, the possibility of photodamage has been investigated in a similar Raman setup previously by Harvey et al. [48]. The results show that using 27.5 mW of 514.5 nm focused laser irradiation, caused minimal damage to cells up to an exposure time of 30s. Therefore, in this current study, the selected integration of 10 s suggests minimal photodamage to the cells using this system.

### Appendix A.6. Cell Viability in Room Temperature DPBS

During the five-point measurements, cells were in DPBS for an hour. The cell viability was tested with a trypan blue assay and pictures were taken under a microscope every 30 min with a 5 × objective, which are shown in Figure A5.

Cell viability after 60 min room temperature DPBS exposure was counted by using Trypan blue assay. Automatic cell counter (Cellometer Auto 1000 Cell Viability Counter, Nexcelom Bioscience Ltd., Manchester, UK) was used and the results are shown in Table A4.

**Table A4:** The cell viabilities (in percentage) obtained.

Sample 1	Sample 2	Sample 3	Average
90.9%	88%	94.1%	91.0%

### Appendix A.7. Monitoring the Change of States of Cells Using Hypoxic Reagent

Experiments were conducted to ensure the cells were under hypoxia after treating them with 100 μM cobalt (II) chloride (CoCl_2_, Sigma-Aldrich, Inc., Poole, UK) for 24 h. A solution of 5 μM ImageIT (concentration suggested by the supplier’s standard procedures, Thermo Fisher Scientific UK Ltd. Hemel Hempstead, UK) was used for monitoring the changes in cell condition. This florescence dye works based on the intracellular oxygen level of the cells which will turn red if the intracellular oxygen level is below 5%. The dye is reversible and is transparent when intracellular oxygen level is increased to 5% or higher.

Cells were seeded into the BioFlux plate two days beforehand allowing them to attach and develop. Standard procedures used for seeding cells into the well plate are described in Section 4.2. Serum-free FluoroBrite with both ImageIT and CoCl_2_ added as supplement were used for treating the cells. Images were taken at the 0 time point and 24 h time point.

Figure A6 shows the example images taken at 0 and 24 h of treatment of one of the microfluidic channels. It can be seen that most of the cells turned hypoxic after 24 h of treatment. Bright field images are also included for locating the cells.

During the Raman measurements, DPBS was used, and therefore it was important to ensure the cells did not switch back to normoxic state during the measurement period.

With 10 s of integration time for each cell and 50 cells per measurement, total DPBS exposure time for one time point was approximately 20–25 min. The fluid in the microfluidic channels was then switched back to the serum-free RPMI containing CoCl_2_ to ensure that the hypoxic condition was maintained. To monitor the effect of DPBS on the cells condition, experiments were performed by exposing cells under hypoxia (treated with CoCl_2_ for 24 h prior) in DPBS for 30 min. ImageIT reagent was used to monitor the condition of cells throughout the experiment.

Figure A7 shows the dark field image of the same area in a channel of cells exposed to DPBS for 0, 10, 20 and 30 min. It is clear that the cells had not switched back into a normoxic state after 30 min.

Although measurements were expected to be completed within 30 min per time point, one further time point at 60 min was imaged. A dark field microscope picture is shown in Figure A8 which shows that the cells were still in a hypoxic state after 60 min of fresh DPBS exposure.

## References

[1] Warburg O. (1956). On the origin of cancer cells. Science.

[2] Hanahan D., Weinberg R.A. (2011). Hallmarks of Cancer: The Next Generation. Cell.

[3] DeBerardinis R.J., Lum J.J., Hatzivassiliou G., Thompson C.B. (2008). The Biology of Cancer: Metabolic Reprogramming Fuels Cell Growth and Proliferation. Cell Metab..

[4] Kaelin W.G., Thompson C.B. (2010). Q&A: Clues from cell metabolism. Nature.

[5] Benjamin D.I., Cravatt B.F., Nomura D.K. (2012). Global Profiling Strategies for Mapping Dysregulated Metabolic Pathways in Cancer. Cell Metab..

[6] Luo X., Cheng C., Tan Z., Li N., Tang M., Yang L., Cao Y. (2017). Emerging roles of lipid metabolism in cancer metastasis. Mol. Cancer.

[7] Baenke F., Peck B., Miess H., Schulze A. (2013). Hooked on fat: The role of lipid synthesis in cancer metabolism and tumour development. Dis. Model. Mech..

[8] Currie E., Schulze A., Zechner R., Walther T.C., Farese R.V. (2013). Cellular fatty acid metabolism and cancer. Cell Metab..

[9] Röhrig F., Schulze A. (2016). The multifaceted roles of fatty acid synthesis in cancer. Nat. Rev. Cancer.

[10] Dubikovskaya E., Chudnovskiy R., Karateev G., Park H.M., Stahl A. (2014). Measurement of long-chain fatty acid uptake into adipocytes. Methods Enzym..

[11] Maier O., Oberle V., Hoekstra D. (2002). Fluorescent lipid probes: Some properties and applications (a review). Chem. Phys. Lipids.

[12] Blanksby S.J., Mitchell T.W. (2010). Advances in Mass Spectrometry for Lipidomics. Annu. Rev. Anal. Chem..

[13] Denbigh J.L., Lockyer N.P. (2014). ToF-SIMS as a tool for profiling lipids in cancer and other diseases. Mater. Sci. Technol..

[14] Gazi E., Harvey T.J., Brown M.D., Lockyer N.P., Gardner P., Clarke N.W. (2009). A FTIR microspectroscopic study of the uptake and metabolism of isotopically labelled fatty acids by metastatic prostate cancer. Vib. Spectrosc..

[15] Gazi E., Gardner P., Lockyer N.P., Hart C.A., Brown M.D., Clarke N.W. (2007). Direct evidence of lipid translocation between adipocytes and prostate cancer cells with imaging FTIR microspectroscopy. J. Lipid Res..

[16] Majzner K., Tott S., Roussille L., Deckert V., Chlopicki S., Baranska M. (2018). Uptake of fatty acids by a single endothelial cell investigated by Raman spectroscopy supported by AFM. Analyst.

[17] Jamieson L.E., Greaves J., McLellan J.A., Munro K.R., Tomkinson N.C.O., Chamberlain L.H., Faulds K., Graham D. (2018). Tracking intracellular uptake and localisation of alkyne tagged fatty acids using Raman spectroscopy. Spectrochim. Acta Part A Mol. Biomol. Spectrosc..

[18] Wei L., Hu F., Shen Y., Chen Z., Yu Y., Lin C.-C., Wang M.C., Min W. (2014). Live-cell imaging of alkyne-tagged small biomolecules by stimulated Raman scattering. Nat. Methods.

[19] Hong S., Chen T., Zhu Y., Li A., Huang Y., Chen X. (2014). Live-cell stimulated Raman scattering imaging of alkyne-tagged biomolecules. Angew. Chem. Int. Ed..

[20] Clemens G., Flower K.R., Gardner P., Henderson A.P., Knowles J.P., Marder T.B., Whiting A., Przyborski S. (2013). Design and biological evaluation of synthetic retinoids: Probing length vs. stability vs. activity. Mol. Biosyst..

[21] Strehle K.R., Cialla D., Rösch P., Henkel T., Köhler M., Popp J. (2007). A reproducible surface-enhanced Raman spectroscopy approach. Online SERS measurements in a segmented microfluidic system. Anal. Chem..

[22] Watson D.A., Brown L.O., Gaskill D.F., Naivar M., Graves S.W., Doorn S.K., Nolan J.P. (2008). A flow cytometer for the measurement of raman spectra. Cytom. Part A.

[23] Dochow S., Krafft C., Neugebauer U., Bocklitz T., Henkel T., Mayer G., Albert J., Popp J. (2011). Tumour cell identification by means of Raman spectroscopy in combination with optical traps and microfluidic environments. Lab Chip.

[24] Perozziello G., Candeloro P., De Grazia A., Esposito F., Allione M., Coluccio M.L., Tallerico R., Valpapuram I., Tirinato L., Das G. (2016). Microfluidic device for continuous single cells analysis via Raman spectroscopy enhanced by integrated plasmonic nanodimers. Opt. Express.

[25] Casabella S., Scully P., Goddard N., Gardner P. (2016). Automated analysis of single cells using Laser Tweezers Raman Spectroscopy. Analyst.

[26] Cancer Research UK Prostate Cancer Incidence Statistics. http://www.cancerresearchuk.org/health-professional/cancer-statistics/statistics-by-cancer-type/prostate-cancer/incidence#heading-Two.

[27] Norrish A.E., Skeaff C.M., Arribas G.L., Sharpe S.J., Jackson R.T. (1999). Prostate cancer risk and consumption of fish oils: A dietary biomarker-based case-control study. Br. J. Cancer.

[28] Dennis L.K., Snetselaar L.G., Smith B.J., Stewart R.E., Robbins M.E.C. (2004). Problems with the assessment of dietary fat in prostate cancer studies. Am. J. Epidemiol..

[29] Giovannucci E., Rimm E.B., Colditz G., Stampfer M.J., Ascherio A., Chute C., Willett W. (1993). A Prospective Study of Dietary Fat and Risk of Prostate Cancer. JNCI J. Natl. Cancer Inst..

[30] Brown M.D., Hart C.A., Gazi E., Bagley S., Clarke N.W. (2006). Promotion of prostatic metastatic migration towards human bone marrow stoma by Omega 6 and its inhibition by Omega 3 PUFAs. Br. J. Cancer.

[31] Šebek J., Pele L., Potma E.O., Benny Gerber R. (2011). Raman spectra of long chain hydrocarbons: Anharmonic calculations, experiment and implications for imaging of biomembranes. Phys. Chem. Chem. Phys..

[32] Wenger R., Kurtcuoglu V., Scholz C., Marti H., Hoogewijs D. (2015). Frequently asked questions in hypoxia research. Hypoxia.

[33] Milosevic M., Warde P., Meńard C., Chung P., Toi A., Ishkanian A., McLean M., Pintilie M., Sykes J., Gospodarowicz M. (2012). Tumor hypoxia predicts biochemical failure following radiotherapy for clinically localized prostate cancer. Clin. Cancer Res..

[34] Furlong S.T., Thibault K.S., Morbelli L.M., Quinn J.J., Rogers R.A. (1995). Uptake and Compartmentalization of Fluorescent Lipid Analogs in Larval Schistosoma-Mansoni. J. Lipid Res..

[35] Mojumdar E.H., Groen D., Gooris G.S., Barlow D.J., Lawrence M.J., Deme B., Bouwstra J.A. (2013). Localization of cholesterol and fatty acid in a model lipid membrane: A neutron diffraction approach. Biophys. J..

[36] Hayes V., Johnston I., Arepally G.M., McKenzie S.E., Cines D.B., Rauova L., Poncz M. (2017). Endothelial antigen assembly leads to thrombotic complications in heparin-induced thrombocytopenia. J. Clin. Invest..

[37] Diebel L.N., Diebel M.E., Martin J.V., Liberati D.M. (2018). Acute hyperglycemia exacerbates trauma-induced endothelial and glycocalyx injury: An in vitro model. J. Trauma Acute Care Surg..

[38] Abumrad N., Coburn C., Ibrahimi A. (1999). Membrane proteins implicated in long-chain fatty acid uptake by mammalian cells: CD36, FATP and FABPm. Biochim. Biophys. Acta Mol. Cell Biol. Lipids.

[39] Williams D.H., Fleming I. (1995). Spectroscopic Methods in Organic Chemistry.

[40] Shimanouchi T. (1972). Tables of Molecular Vibrational Frequencies.

[41] Brown M.D., Hart C., Gazi E., Gardner P., Lockyer N., Clarke N. (2010). Influence of omega-6 PUFA arachidonic acid and bone marrow adipocytes on metastatic spread from prostate cancer. Br. J. Cancer.

[42] Rezende L.P., Galheigo M.R.U., Landim B.C., Cruz A.R., Botelho F.V., Zanon R.G., Góes R.M., Ribeiro D.L. (2019). Effect of glucose and palmitate environment on proliferation and migration of PC3-prostate cancer cells. Cell Biol. Int..

[43] Kim S., Yang X., Yin A., Zha J., Beharry Z., Bai A., Bielawska A., Bartlett M.G., Yin H., Cai H. (2019). Dietary palmitate cooperates with Src kinase to promote prostate tumor progression. Prostate.

[44] Yang Q., Wang S., Ji Y., Chen H., Zhang H., Chen W., Gu Z., Chen Y.Q. (2017). Dietary intake of n-3 PUFAs modifies the absorption, distribution and bioavailability of fatty acids in the mouse gastrointestinal tract. Lipids Health Dis..

[45] Holman R.T. (1958). Essential Fatty Acids. Nutr. Rev..

[46] Bensaad K., Favaro E., Lewis C.A., Peck B., Lord S., Collins J.M., Pinnick K.E., Wigfield S., Buffa F.M., Li J.L. (2014). Fatty acid uptake and lipid storage induced by HIF-1α contribute to cell growth and survival after hypoxia-reoxygenation. Cell Rep..

[47] Wu D., Yotnda P. (2011). Induction and Testing of Hypoxia in Cell Culture. J. Vis. Exp..

[48] Harvey T.J., Faria E.C., Henderson A., Gazi E., Ward A.D., Clarke N.W., Brown M.D., Snook R.D., Gardner P. (2008). Spectral discrimination of live prostate and bladder cancer cell lines using Raman optical tweezers. J. Biomed. Opt..

